# With PBDB-T as the Donor, the PCE of Non-Fullerene Organic Solar Cells Based on Small Molecule INTIC Increased by 52.4%

**DOI:** 10.3390/ma13061324

**Published:** 2020-03-14

**Authors:** Weifang Zhang, Zicha Li, Suling Zhao, Zheng Xu, Bo Qiao, Dandan Song, S. Wageh, Ahmed Al-Ghamdi

**Affiliations:** 1 Key Laboratory of Luminescence and Optical Information, Ministry of Education, Beijing Jiaotong University, Beijing 100044, China; 17121599@bjtu.edu.cn (W.Z.); 16118446@bjtu.edu.cn (Z.L.); zhengxu@bjtu.edu.cn (Z.X.); boqiao@bjtu.edu.cn (B.Q.); ddsong@bjtu.edu.cn (D.S.); 2 Department of Physics, Faculty of Science, King Abdulaziz University, Jeddah 21589, Saudi Arabia; wageh1@yahoo.com (S.W.); aghamdi90@hotmail.com (A.A.-G.); 3 Physics and Engineering Mathematics Department, Faculty of Electronic Engineering, Menoufia University, Menouf 32952, Egypt

**Keywords:** polymer solar cells (PSCs), non-fullerene small molecule acceptor, synthesize easily, strong and wide infrared absorption, carrier mobility, carrier transportation and extraction

## Abstract

At present, most high-performance non-fullerene materials are centered on fused rings. With the increase in the number of fused rings, production costs and production difficulties increase. Compared with other non-fullerenes, small molecule INTIC has the advantages of easy synthesis and strong and wide infrared absorption. According to our previous report, the maximum power conversion efficiency (PCE) of an organic solar cell using PTB7-Th:INTIC as the active layer was 7.27%. In this work, other polymers, PTB7, PBDB-T and PBDB-T-2F, as the donor materials, with INTIC as the acceptor, are selected to fabricate cells with the same structure to optimize their photovoltaic performance. The experimental results show that the optimal PCE of PBDB-T:INTIC based organic solar cells is 11.08%, which, thanks to the open voltage (V_OC_) increases from 0.80 V to 0.84 V, the short circuit current (J_SC_) increases from 15.32 mA/cm^2^ to 19.42 mA/cm^2^ and the fill factor (FF) increases from 60.08% to 67.89%, then a 52.4% improvement in PCE is the result, compared with the devices based on PTB7-Th:INTIC. This is because the PBDB-T:INTIC system has better carrier dissociation and extraction, carrier transportation and higher carrier mobility.

## 1. Introduction

Solar cells based organic materials have attracted more and more attention as promising candidates for solving problems of the global energy shortage and climate change, because of the advantages of their lighter weight, lower production costs, simple manufacturing processes, easy preparation of flexible devices and easy mass production [1,2,3,4,5,6,7]. Fullerene derivatives are widely used as acceptor materials for organic solar cells because of their excellent electron transport property and easy to form good bulk heterojunctions with polymer or small molecular donors [8,9]. The constant design and improvement of donor materials by researchers, and the efficiency of the bulk heterojunction organic solar cells of the polymer-fullerene system, have reached more than 10% [10]. However, fullerene materials still have some disadvantages, including their high synthetic costs [11], the weak and limited absorption of incident solar light [11,12,13,14] and the instable morphology due to the fullerene molecule diffusion and aggregation in the thin film over time [15,16]. Even some small molecular materials have been gradually synthesized as new acceptors, in order to overcome these shortcomings. Compared with fullerenes, non-fullerenes have obvious advantages, such as the easy adjustment of their molecular energy levels, their superior light absorption properties and potentially low-cost synthetic processes [8,14,16,17].

Non-fullerene organic solar cells have made rapid progress in recent years. Especially in the past three years, the research on non-fullerene acceptors based on the acceptor-donor-acceptor (A-D-A) structure have made breakthrough progress [5,18]. At present, there are two strategies to improve the near-infrared (NIR) absorption of non-fullerene materials. One way is extending the π-system and many successes have been achieved [8,19,20,21]. The other way is enhancing the electron-donating capability of the central D core [22,23]. However, most NIR-absorbing high-performance non-fullerene materials are centered on fused rings. With the increase in the number of fused rings, the production costs and production difficulties increase correspondingly. Therefore, the development of central D cores that are easy to be synthesized is still a challenge which needs to be conquered at present [24]. The reported 2,2’-((2Z,2’Z)-((6,6’-(5,10,10-tris(2-ethylhexyl)-5,10-dihydroindeno[1,2-b]indole-2,7-diyl)bis(2-octylthieno[3,4-b]thio-phene-6,4-diyl))bis(methanylylidene))bis(5,6-difluoro-3-oxo-2,3-dihydro-1H-indene-2,1-diylidene))dimalononitrile (INTIC) has used indeno[1,2-b]indole (IN) as the D cores [24]. IN was reported as not only having an extended π-system with a good electron-donating capability deriving from its indole nitrogen atom, but it could also be synthesized easily by one step from available commercial materials [24,25,26]. This means that INTIC is easily synthesized compared to other non-fullerene acceptors. In our previous report, Poly[4,8-bis(5-(2-ethylhexyl)thiophen-2-yl)benzo[1,2-b;4,5-b’]dithiophene-2,6-diyl-alt-(4-(2-ethylhexyl)-3-fluoro-thieno[3,4-b]thiophene-)-2-carboxylate-2-6-diyl)] (PTB7-Th) was selected as the donor, INTIC as the acceptor, and the ITO/ZnO/PTB7-Th:INTIC/MoO_3_/Ag inverse device structure was adopted, and the maximum power conversion efficiency (PCE) was 7.27% after optimization [24]. However, the short circuit current (J_SC_) and filling factor (FF) of this device were not high due to its low carrier separation, low carrier mobility and poor film crystallinity. In organic photovoltaic devices, the performance of the device varies greatly when the same acceptor is combined with different donors. The factors that affect the value of J_SC_ are the absorption of sunlight by the active layer, generation of excitation, diffusion, separation, and charge transportation and collection. The open-circuit voltage (V_OC_) is determined by the HOMO (highest occupied molecular orbital) level of the donor and the LUMO (lowest unoccupied molecular orbital) level of the acceptor according to the reference [27,28]. Through the selection of different donors, the acceptor can be matched with the donor with different LUMO and HOMO. In addition, the energy barrier between the donor and acceptor will facilitate the carrier separation and block the carrier recombination to increase J_SC_ and FF.

In this article, we continue to explore the INTIC system and choose different donors with different energy levels to fabricate organic photovoltaics (OPVs) and to improve the performance of devices based on INTIC as the acceptor. It is proven that the LUMO barrier between the donor and the acceptor and the film morphology are very important to the carrier collection of OPVs, with INTIC as the acceptor. 

## 2. Materials and Methods

### 2.1. Materials

The small-molecule acceptor INTIC was synthesized in this work. The donor material PTB7(PTB7: Poly [[4,8-bis[(2-ethylhexyl)oxy]benzo[1,2-b:4,5-b’]dithiophene-2,6-diyl][3-fluoro-2-[(2-ethylhexyl)carbonyl]thieno[3,4-b]thiophenediyl]]) and PTB7-Th were purchased from 1-materials. The donor PBDB-T(PBDB-T: Poly[(2,6-(4,8-bis(5-(2-ethylhexyl)thiophen-2-yl)-benzo[1,2-b:4,5-b’]dithiophene))-alt-(5,5-(1′,3′-di-2-thienyl-5′,7′-bis(2-ethylhexyl)benzo[1′,2′-c:4′,5′-c’]dithiophene-4,8-dione)]) and PBDB-T-2F(PBDB-T-2F: Poly[(2,6-(4,8-bis(5-(2-ethylhexyl-3-fluoro)thiophen-2-yl)-benzo[1,2-b:4,5-b’]dithiophene))-alt-(5,5-(1′,3′-di-2-thienyl-5′,7′-bis(2-ethylhexyl)benzo[1′,2′-c:4′,5′-c’]dithiophene-4,8-dione)]) were obtained from Solarmer Materials Inc in Beijing, China.

The zinc oxide precursor solution was prepared from a mass ratio of zinc acetate dihydrate: ethanolamine: 2-methoxyethanol = 0.25 g: 0.07 g: 2.288 g, and the above materials were purchased from Sigma-Aldrich in Shanghai, China.

### 2.2. Device Fabrication and Characterizations

**Figure 1:**
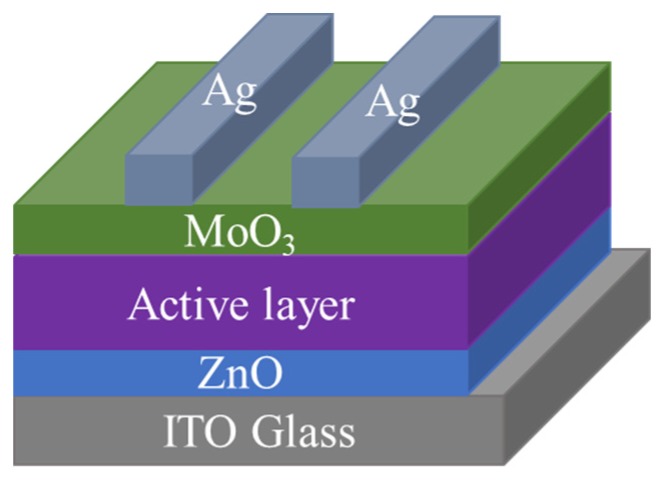
Schematic devices architecture of the n-i-p OPVs, constructed by ITO/ZnO/Active Layer/MoO_3_/Ag.

In order to evaluate the devices’ performance, organic solar cells in this article were fabricated in an inverted architecture of ITO/ZnO/Active Layer/MoO_3_/Ag, as shown in Figure 1. Before use, ITO substrates with a square resistance of 15 Ω/cm^2^ were ultrasonically cleaned with the cleaning agent, deionized water and anhydrous alcohol for 30 min each in an ultrasonic cleaning machine. Then, ITO substrates were placed face up in a plasma surface treatment apparatus for 90 s, before the spin-coating of ZnO to improve the hydrophilicity and surface work function of the ITO surface. The ZnO precursor solution was spin-coated at a speed of 4000 rpm/min for 40 s on the ITO surface. The coated substrates were then placed on the annealing platform and annealed at 180 ℃ for 30 min in the fume hood, to improve the crystallization of ZnO layer. Donor and acceptor were dissolved in chloroform (CF) at a ratio of 1:1.5, at a total concentration of 10 mg/mL. Before preparing the active layer, 1% 1-chloronaphthalene (CN) was added to the configured blend solution as the additive solvent, and finally spin-coated at 2000 rpm/min for 40 s. Then, they were placed in a darkened petri dish and dried in a vacuum for one hour. Then, 10 nm MoO_3_ layer and 100 nm Ag layer were sequentially evaporated through a shadow mask under a vacuum of 5 × 10^−4^ Pa, after the active layer was dried.

The UV-Vis absorption spectra were acquired on a Shimadzu UV-3101 PC spectrometer, which was purchased from Shimazu company (Kyoto, Japan). The surface morphology characteristics were measured by atomic force measurement (AFM, PSIA XE-100). We did this test in Tsinghua university, China. Grazing incidence X-ray diffraction (GIXRD) was measured in Beijing synchrotron radiation laboratory. Current density-voltage (J-V) curves were measured in a Keithley 2400 Source Measure Unit, purchased from Keithley, Beijing, China. Photocurrent was measured in an Air Mass 1.5 Global (AM 1.5 G) solar simulator (Class AAA solar simulator, sirius-SS150A-D), purchased from Beijing, China, with an irradiation intensity of 100 mW/cm^2^, which was calibrated by a silicon solar cell.

## 3. Results and Discussion

**Figure 2:**
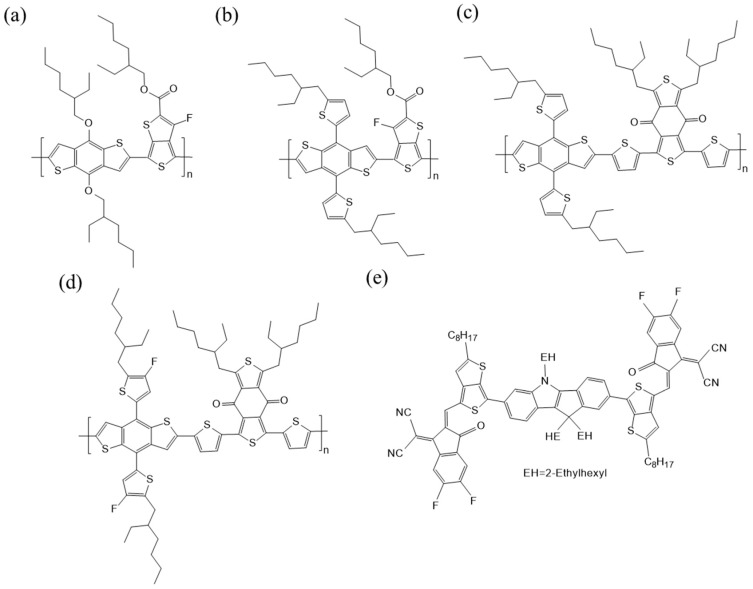
Chemical structures of (**a**) PTB7, (**b**) PTB7-Th, (**c**) PBDB-T, (**d**) PBDB-T-2F and (**e**) INTIC.

**Figure 3:**
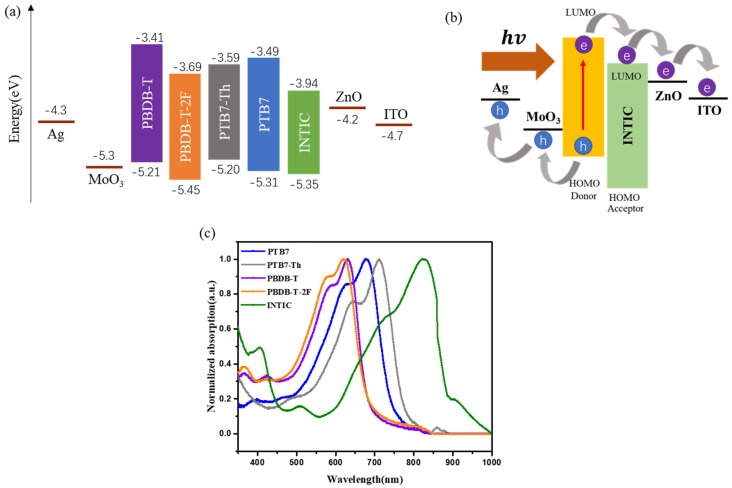
(**a**) Energy level alignment of materials used in the active layer; (**b**) Diagram of charge separation and transportation; (**c**) UV–Vis absorption spectra of PTB7, PTB7-Th, PBDB-T, PBDB-T-2F and INTIC pure film respectively.

In our previous study, we have reported that the optimal PCE of PTB7-Th:INTIC based organic solar cell was 7.27%. In this work, PTB7-Th:INTIC based cell is used as a control device, other polymers PTB7, PBDB-T and PBDB-T-2F as the donors, with INTIC as the acceptor, are selected to fabricate cells with a same structure to optimize their photovoltaic performance. The cell and the chemical structures, the energy band of used materials, as well as the brief process of charge separation and transportation, as shown in Figure 1, Figure 2, Figure 3a and Figure 3b, respectively. Firstly, the UV–Vis absorption spectrum of the films of pure PTB7, PTB7-Th, PBDB-T, PBDB-T-2F and INTIC are measured, respectively, as shown in Figure 3c. The film of INTIC shows an intense and wider absorption band in 650–900 nm. For films of PTB7, PTB7-Th, PBDB-T and PBDB-T-2F, their absorption band is located in 550–730 nm, 580–750 nm, 500–650 nm and 500–650 nm, respectively. Although the HOMO level and LUMO level of PBDB-T and PBDB-T-2F are located at different levels, shown in Figure 3a, their absorption spectra are almost the same, except a little blue-shift of PBDB-T-2F, because of their similar band gaps. According to these results, it finds that the absorption spectra of four polymers are complementary to that of INTIC, especially the strong infrared absorption of INTIC, and their energy levels are matched to the energy level of the small molecule INTIC.

**Figure 4:**
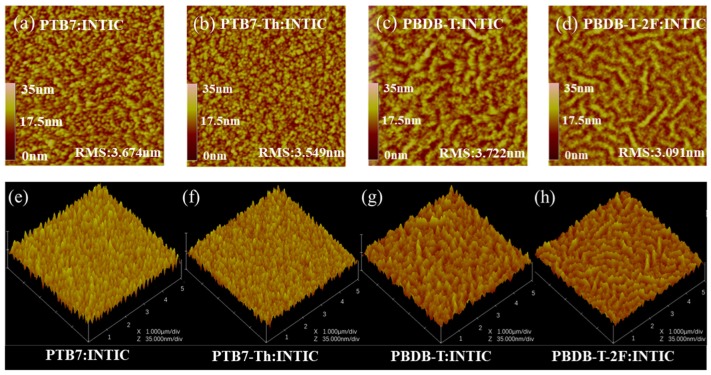
AFM images of the active layer with (**a** and **e**) PTB7:INTIC; (**b** and **f**) PTB7-Th:INTIC; (**c** and **g**) PBDB-T:INTIC; (**d** and **h**) PBDB-T-2F:INTIC.

**Figure 5:**
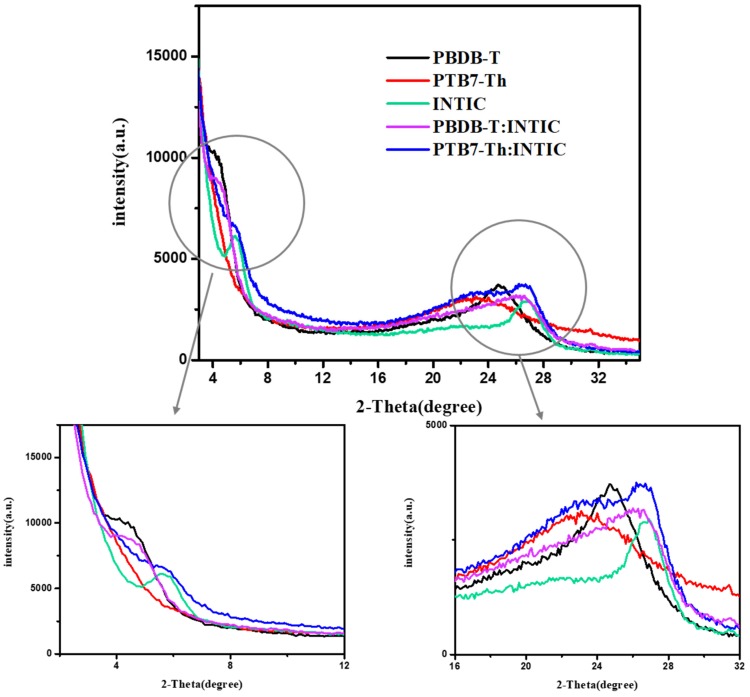
One-dimensional out-of-plane GIXRD of PTB7-Th, PBDB-T, INTIC pure film and PTB7-Th:INTIC, PBDB-T:INTIC mixed film.

Firstly, the morphology of four active layers as PTB7:INTIC, PTB7-Th:INTIC, PBDB-T:INTIC and PBDB-T-2F:INTIC spin-coated on ZnO film are presented by tapping-mode AFM images of films, as shown in Figure 4, for investigating the blend film quality. Among them, the blend film with PBDB-T:INTIC shows a little surface roughness, with a root mean square (RMS) value of about 3.722 nm, as shown in Figure 4c. It images that the PBDB-T:INTIC mixed film has a good and uniform crystallinity. Additionally, the grain boundary in the PBDB-T:INTIC mixed film is more obvious compared to the other three films. In addition, as the sample, the crystallization of PBDB-T single-layer film, PTB7-Th single-layer film, INTIC single-layer film and their blended films by using the GIXRD one-dimensional point detection mode, as shown in Figure 5. PBDB-T film shows two clear GIXRD peaks at 2θ of 4° and 24.7°, which still shows in GIXRD of the blend film PBDB-T:INTIC, except the peak of INTIC around 26.2°. It gives the evidence that both PBDB-T and INTIC have a good crystallinity in the blend film, which is favored to the hole and electron transporting separately. In film of pure PTB7-Th shows only a wide peak around 22.5°. The wide peak also is observed in the mixed film of PTB7-Th: INTIC, which means that PTB7-Th crystallinity in the pure PTB7-Th film and in the mixed film of PTB7-Th: INTIC is not good enough, as even INTIC in the blend of PTB7-Th: INTIC shows its clear GIXRD peaks. The poor crystallinity of PTB7-Th will influence the hole transportation and decreases the hole mobility in the blend film. 

**Figure 6:**
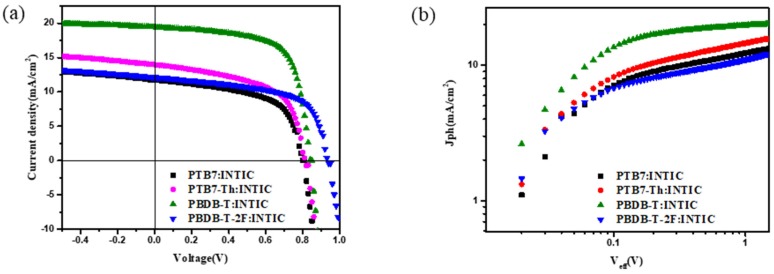
(**a**) J-V characteristics of the PSCs with different donors under irradiation of 100 mW/cm^2^ with reverse scan; (**b**) J_ph_-V_eff_ characteristics of the devices with different donors.

Different devices with the same structure of ITO/ZnO/Active Layer/MoO_3_/Ag are prepared, in which the active layers are PTB7:INTIC, PTB7-Th:INTIC, PBDB-T:INTIC and PBDB-T-2F:INTIC, respectively. The preliminary exploration of device performance through the J-V test is carried out. Figure 6a shows the J-V characteristics of different prepared devices under AM1.5G illumination, with the intensity of 100 mW/cm^2^. The photovoltaic performance parameters of fabricated solar cells were induced and shown in Table 1, in which the values of each parameter are the average date of four devices. The control device with PTB7-Th:INTIC with 1% CN additive exhibits a V_OC_ of 0.811 V, J_SC_ of 13.9544 mA/cm^2^ and FF of 57.534%, which results in a PCE of 6.528%. The devices using PTB7: INTIC and PBDB-T-2F: INTIC as active layers have unsatisfactory device performance, with PCE of 5.260% and 6.811%, respectively. When the donor is polymer PBDB-T, the device shows a remarkable high PCE of 10.655% with V_OC_ of 0.847 V, J_SC_ of 18.995 mA/cm^2^ and FF of 66.245%. In addition, its shunt resistance (R_sh_) increases from 8461.614 Ω/cm^2^ to 15863.343 Ω/cm^2^, and its series resistance (R_s_) decreases from 181.171 Ω/cm^2^ to 139.132 Ω/cm^2^, compared with that of the control device. The low R_s_ value and high R_sh_ value also mean that the transportation and extraction of charge in PBDB-T:INTIC system is better than that of PTB7-Th:INTIC system. The experimental results show that the highest PCE of PBDB-T:INTIC is 11.08% (J_SC_: 19.42 mA/cm^2^, V_OC_: 0.84V, FF: 67.89%) and increases about 52.4% compared with the highest PCE 7.27% reported of PTB7-Th:INTIC. It implies that PBDB-T:INTIC can suppress the leakage current effectively under reverse bias and promote the carrier transport in the PBDB-T:INTIC blend layer, which results in an increased J_SC_ and FF compared with the control cells.

The HOMO levels of PBDB-T and PTB7-Th are similar, as shown in Figure 3a, but the LUMO level of PBDB-T is higher than the LUMO of PTB7-Th. Scharber’s research group found that donor materials with different HOMO levels and fixed acceptor materials were used to make devices, and obtained the empirical formula for the V_OC_ of organic solar cells:

(1) $$V_{OC}=\frac{1}{q}\left(\left|E_{HOMO}\right|-\left|E_{LUMO}\right|\right)-0.3eV$$

Among them, $E_{HOMO}$ is the HOMO level of the donor material, $E_{LUMO}$ is the LUMO level of the acceptor, and 0.3 eV is the empirical value [29]. Equation (1) gives us a reference to evaluate the V_OC_ which is also affected by the interface modification layer on both sides of the active layer, the electrode work function, non-radiative recombination loss, and the driving force of charge separation [30]. According to Equation (1), V_OC_ of two cells with the same structure and modification will be same because of the closing HOMO level for two donors. However, V_OC_ of the cell based on PBDB-T:INTIC is little higher than that of PTB7-Th:INTIC. It implies that the higher LUMO level of PBDB-T plays a rule to the contribution of photovoltaic performance, for which the higher LUMO level is beneficial to the charge separation and blocking electrons back from INTIC to PBDB-T. Therefore, we can consider that the exciton dissociation and charge collection is higher in PBDB-T: INTIC system than that in PTB7-Th:INTIC system. Starting from this aspect, we analyze the reasons for the poor performance of PTB7:INTIC and PBDB-T-2F:INTIC devices. PTB7 has a close LUMO level with PBDB-T and a lower HOMO level than PBDB-T. Compared with the PBDB-T: INTIC system, V_OC_ of PTB7: INTIC system is larger, due to the low HOMO level of PTB7. However, its HOMO level closes to that of INTIC, holes in PTB7 relax easily to INTIC by thermal relaxation, which results in a higher carrier recombination and a bad carrier separation. So, it causes a decrease in the PTB7:INTIC system. PBDB-T-2F has a low HOMO level, which makes the PBDB-T-2F:INTIC system have a high V_OC_. The HOMO level of PBDB-T-2F is lower than the HOMO level of INTIC, which results in holes transfer to INTIC easily and hinders the transport of holes. Its lower LUMO level than that of PTB7-Th implies a low driving force for exciton dissociation and leads to a low J_SC_. 

To make a further argument for the above discussion, the photocurrent density (J_ph_) versus the effective applied voltage (V_eff_) was measured, as shown in Figure 6b. J_ph_ is defined as J_ph_ = J_L_ − J_D_, where J_L_ and J_D_ is the current density under the illumination and the dark. Additionally, V_eff_ is defined as V_eff_ = V_0_ − V_bias_, where V_0_ is the voltage when photocurrent is equal to zero and V_bias_ is the external applied voltage bias [31]. As can be seen from Figure 6b, for devices based on PBDB-T:INTIC system, J_ph_ reaches its saturation faster than other devices, and a higher J_ph_ is obtained at low V_eff_. It means that the photogenerated carrier extraction and charge collection were enhanced, and the carrier recombination was reduced in this system. However, for the other three devices, J_ph_ does not reach its saturation, even under a higher external voltage. It means that there are more excitons recombined and the carrier extractions are difficult. This supports our previous reasoning. Then, we calculated the carrier collection efficiency P of different devices according to the formula of P = J_ph-sc_/J_sat_, in which J_ph-sc_ and J_sat_ stand for J_ph_ and saturation current density under short-circuit condition, respectively [32]. The photocurrent at the highest external voltage is used as J_sat_ for the other three devices. The calculated P is 88.40%, 89.10%, 96.80% and 86.33% for PTB7:INTIC, PTB7-Th:INTIC, PBDB-T:INTIC and PBDB-T-2F:INTIC system devices, respectively. A highest P is reached for a PBDB-T:INTIC based device, which proves again that PBDB-T:INTIC based device has a good carrier separation and collection. So, it is concluded that PBDB-T:INTIC based devices have the best performance among these four systems. 

**Figure 7:**
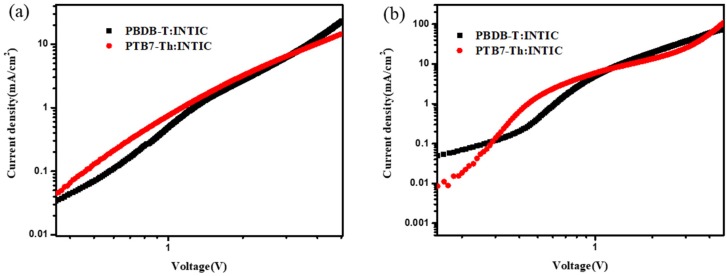
(**a**) J-V curves of ITO/ZnO/Active Layer/Al to measure the electron mobility of PTB7-Th:INTIC and PBDB-T:INTIC; (**b**) J-V curves of ITO/PEDOT:PSS/Active Layer/Au to measure the hole mobility of PTB7-Th:INTIC and PBDB-T:INTIC.

In order to further compare the photovoltaic performance of PTB7-Th:INTIC and PBDB-T:INTIC based cells, the carrier transport in these two active layers are explored by the method of space limiting current (SCLC), to confirm the contribution of the carrier transport to PCE. In organic solar cells, the SCLC method is a commonly used method for measuring the mobility of organic electrons and holes. However, the premise of the SCLC method is that the injection at both ends of the electrode must be ohmic injection, that is, the carrier is injected into the device under dark conditions without affecting the movement of the carrier. Generally, an organic solar cell does not form an ohmic contact between the acceptor material and the cathode, and an interface modification is required to change the work function, thereby forming an ohmic contact between the active layer and the cathode. The SCLC is described by the Mott–Gurney equation:

(2) $$J=\frac{9}{8}\varepsilon_{r}\varepsilon_{0}\mu \frac{V^{2}}{L^{3}}$$

where μ is the carrier mobility. $\varepsilon_{r}$ is the dielectric constant, generally is 3 for common organic material. $\varepsilon_{0}$ is the vacuum permittivity. V is the applied voltage and *L* is the film thickness [3]. In this study, the electron-only device ITO/ZnO/Active Layer/Al and the hole-only device ITO/PEDOT:PSS/Active Layer/Au were prepared, to analyze the electron and hole transport of the active layer with PTB7-Th:INTIC and PBDB-T:INTIC, respectively. The thickness L of all active layers is fixed at about 70 nm. The measured J-V curves are shown in Figure 7. Then, the electron mobility ($\mu_{e}$) and hole mobility ($\mu_{h}$) of the active layers are determined according the Equation (2) to be 2.31 × 10^−3^ cm^2^/Vs and 4.83 × 10^−4^ cm^2^/Vs for PTB7-Th:INTIC, and 6.02 × 10^−3^ cm^2^/Vs and 7.72 × 10^−4^ cm^2^/Vs for PBDB-T:INTIC, respectively. Therefore, it is concluded that, compared with the PTB7-Th:INTIC system, the PBDB-T:INTIC system has a significant increase in electron and hole mobility, which means that the charge transport is enhanced evidently in this system, and this then results in an improvement of J_SC_, FF, as well as PCE of the cells, based on PBDB-T:INTIC system. 

## 4. Conclusions

In this paper, the INTIC system is further optimized. PTB7-Th, PBDB-T, PTB7 and PBDB-T-2F are selected as donors, respectively. The experimental results show that the PCE is 11.08% when PBDB-T:INTIC is the active layer. It is observed that the V_OC_ increases from 0.80 V to 0.84 V, the J_SC_ increases from 15.32 mA/cm^2^ to 19.42 mA/cm^2^ and the FF increases from 60.08% to 67.89%, resulting in a PCE with 52.4% improvement, compared with devices based PTB7-Th:INTIC system. According to the investigation, it is found that the carrier dissociation in PBDB-T:INTIC cell is highest among four kinds of cells due to the higher LUMO level of PBDB-T and good crystallinity of the blend film. The blend film with PBDB-T:INTIC shows a little surface roughness, with an RMS value of about 3.722 nm. Additionally, both the peaks belong to PBDB-T and INTIC are detected respectively in the GIXRD of the blend film of PBDB-T:INTIC. The results, according to the SCLC measurements, show that PBDB-T:INTIC mixed film has a relative higher electron and hole mobility compared with that of PTB7-Th:INTIC blend film. It is favorable for the carrier transportation and extraction. Therefore, a highest PCE of PBDB-T:INTIC based cell has been achieved among four kinds of prepared cells. Additionally, it is concluded that the suitable donor is very important for the acceptor INTIC, a low cost and strong near-infrared absorption capacity acceptor, to fabricate cells with a high photovoltaic performance.

## Figures and Tables

**Table 1:** Summary of OPVs devices’ performance of PTB7:INTIC, PTB7-Th:INTIC, PBDB-T:INTIC and PBDB-T-2F:INTIC fabricated from CF with CN as an additive.

D:A	V_OC_ (V)	J_SC_ (mA/cm^2^)	FF (%)	PCE (%)	R_s_ (Ω/cm^2^)	R_sh_ (Ω/cm^2^)
PBDB-T:INTIC	0.847	18.995	66.245	10.655	139.132	15863.343
PBDB-T-2F:INTIC	0.926	12.184	60.346	6.811	176.240	10190.458
PTB7-Th:INTIC	0.811	13.954	57.534	6.528	181.171	8461.614
PTB7:INTIC	0.801	11.409	57.561	5.260	231.350	9103.349
